# The Natural Defense: Anti-Aging Potential of Plant-Derived Substances and Technological Solutions Against Photoaging

**DOI:** 10.3390/ijms26168061

**Published:** 2025-08-20

**Authors:** Martyna Nowak-Perlak, Marta Olszowy, Marta Woźniak

**Affiliations:** Department of Clinical and Experimental Pathology, Division of General and Experimental Pathology, Wroclaw Medical University, 50-368 Wroclaw, Poland

**Keywords:** plant compounds, photoaging, anti-aging

## Abstract

Photoaging is a multifactorial and progressive skin aging process primarily triggered by prolonged exposure to ultraviolet (UV) radiation. This condition leads to both structural and functional impairments in the skin, including the formation of wrinkles, loss of elasticity, pigmentation irregularities, and an elevated risk of skin malignancies. At the core of photoaging is the accumulation of reactive oxygen species (ROS), which generate oxidative stress, initiate chronic inflammation, cause DNA damage, and accelerate the breakdown of the extracellular matrix—largely through the activity of matrix metalloproteinases (MMPs). The review provides a comprehensive analysis of various natural substances, including antioxidants, anti-inflammatory agents, photoprotective compounds, and emerging regenerative treatments, based on in vitro and in vivo research. Special emphasis is placed on natural substances, including polyphenols, cannabinoids, carotenoids, retinoids, and vitamins, highlighting their potential in preventing and treating photoaging. This review aims to present a detailed, evidence-based overview of photoaging mechanisms and innovative approaches to mitigate its effects.

## 1. Introduction

The skin is the largest organ of the body and acts as a physical and immunological barrier, preventing water loss, protecting against mechanical damage, environmental insults, and microbial invasion. It also contributes to thermoregulation and serves as a sensory organ through its extensive neural network [1,2,3]. Structurally, skin consists of the epidermis and dermis supported by the subcutaneous tissue [1,4,5,6]. The stratum corneum (SC), the highest layer of the skin, is composed of corneocytes (dead keratinized cells encased in a lipid matrix). Keratinocytes, the epidermis’s most prominent cell type, are produced in its basal layer. They are programmed to move to the skin’s surface. The process, which takes around four weeks, involves the keratinocytes changing their shape and beginning to create various keratins, growth factors, and cytokines. The dermis is a thick layer (1–4 mm thick) made up of an extracellular collagen matrix that serves as structural support for the epidermis as well as a source of nutrition for the viable epidermis. Its main goals are to regulate temperature, hydrate, and support the viable epidermis. In addition to the vascular bed, this layer contains a variety of cell types. Fibroblasts, a prominent cell type in the dermis, are responsible for producing dermal collagen. The basal membrane creates a close link between the dermis and epidermis [1,4,5,7]. To facilitate smooth gliding between skin and muscle, the subcutaneous fascia extracellular matrix (ECM) is loaded with an aqueous matrix including glycosaminoglycans such as hyaluronan. Fibroblasts and fasciacytes help produce ECM in the fascia. Fibroblasts secrete fibrous matrix components such as fibrillin, elastin, fibronectin, and collagens I and III, while fasciacytes create hyaluronan [1,8,9].

As skin aging affects not only physiological function and health but also aesthetic appearance and quality of life, there is growing scientific and commercial interest in effective strategies to protect and rejuvenate the skin cells and components. Among these, naturally derived bioactive substances have gained significant attention due to their antioxidant, anti-inflammatory, and photoprotective properties.

Skin aging is a complex process that includes reduced physiological integrity, diminished function, and increased vulnerability to risk factors and sickness. These changes arise naturally with advancing age and are influenced by multiple diverse factors, which occur in two main pathways—intrinsic and extrinsic. Intrinsic aging is linked to genetics, sickness, programmed aging, and cellular senescence, which are all produced by endogenous oxidative stress and cell damage. Extrinsic aging is caused by environmental variables such as smoking, alcohol intake, ultraviolet (UV) radiation, and pollution, which produce reactive oxygen species (ROS), resulting in DNA damage and cellular breakdown. The environmental stressors generate ROS, which contribute to DNA damage, protein degradation, and breakdown of the extracellular matrix (ECM), thereby accelerating the visible signs of skin aging.

The leading cause of skin aging is chronic and prolonged exposure to ultraviolet (UV) radiation, also referred to as photoaging. This process is related to three primary factors: the amount of ultraviolet (UV) radiation on the skin, the amount of melanin in the skin, and the physiological propensity to photoaging [10]. The characteristics of early aging of the skin include angiogenesis, immunological and inflammatory responses, deep wrinkles, delayed wound healing, hyperpigmentation, loss of suppleness, epidermal thickening, susceptibility to cancer, and formation of ROS [11,12].

As UV radiation is the main contributing factor to photoaging, it induces numerous harmful effects in the skin. Overexposure to UV radiation increases the formation of ROS, which at higher concentrations can damage the main proteins that make up the skin, collagen and elastin. Importantly, UVA (range 320–400 nm) radiation has a negative effect on the epidermal keratinocytes and dermal fibroblast and induces long-term changes. UVA radiation leads to the activation of various oxidative stress pathways. The matrix metalloproteinases (MMPs) that are upregulated as a result are thought to drive extrinsic aging (or photoaging) of the skin, degrading type I and type III collagen in the dermis to form fine and coarse wrinkles. Changes arising as a result of UVB (range 280–320 nm) radiation are visible mainly within the epidermis but it also penetrates the upper part of the dermis. UVB radiation directly damages DNA, characteristically introducing bulky adducts like cyclobutene pyrimidine dimers (CPDs) and double-stranded breaks (DSBs) [13,14]. A characteristic feature of the skin affected by photoaging mainly by UVA radiation is the presence of solar elastosis in the dermis. Solar elastose is a dystrophic elastic material that is formed as a result of a cycle of processes leading to the degradation of elastic fibers, followed by the formation of the (ECM) and its reconnection into a structure other than its original one. [13]

At the molecular level, UV radiation triggers the overproduction of reactive oxygen species (ROS), which can directly damage biological macromolecules, induce immune responses and degrade various tissue components.

**Figure 1:**
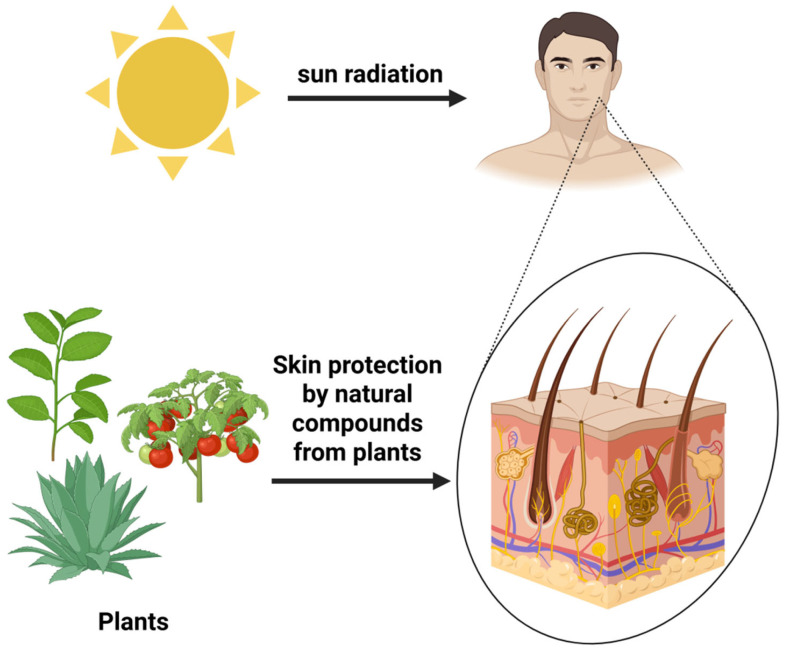
Natural bioactive compounds in photoaging prevention and treatment.

Two main approaches focus on preventing UV-induced skin damage and photoaging. One is focused on blocking UV radiation from entering the skin to avoid UV radiation penetration. For example, it is recommended to cover the body with long sleeves and stay in shaded areas. Interestingly, many substances of natural and synthetic origin have the ability to neutralize ROS, which has anti-aging properties (Figure 1) [15].

The aim of this review is to present and critically assess the current state of research on plant-based and naturally occurring substances like vitamins with anti-photoaging potential. Particular emphasis is placed on their mechanisms of action, including ROS neutralization, inhibition of matrix metalloproteinases (MMPs), support of skin barrier integrity, and modulation of inflammatory responses. This review also explores the application potential of these substances in both therapeutic and cosmetic dermatology, offering an evidence-based perspective on their role in preventing and reversing UV-induced skin damage.

## 2. Methodology

A literature search was conducted using the PubMed database to identify relevant publications on the role of plant compounds in photoaging and anti-aging. The search was performed using the following keywords: photoaging, plant compounds, and anti-aging. Articles were selected based on their relevance to the topic, with a focus on studies discussing the mechanisms, efficacy, and potential applications of plant-derived compounds in preventing or mitigating photoaging. Inclusion criteria included peer-reviewed articles published in English that addressed experimental, clinical, or review data on plant compounds with anti-photoaging effects. Publications not directly related to the core topic or lacking scientific rigor were excluded.

## 3. Bioactive Substances in Photoaging Prevention and Treatment

### 3.1. Polyphenols

#### 3.1.1. Phenolic Acids

Phenolic acids, such as caffeic acid, ferulic acid, gallic acid and sinapic acid, exhibit strong antioxidant and anti-inflammatory properties, making them effective agents in photoprotection. Caffeic acid acts as a prooxidant in certain conditions, mobilizing endogenous copper and inducing oxidative DNA damage, contributing to its anticancer properties [16]. It prevents photoaging by inhibiting UV-induced MMP-1 expression through suppression of histone acetyltransferases, which regulate pro-inflammatory gene expression [17]. Additionally, caffeic acid has demonstrated protective effects against UVB-induced oxidative damage in human fibroblasts by reducing ROS production and enhancing cellular defense mechanisms [18]. In human skin fibroblasts exposed to UVB radiation, it inhibits MMP-1 production and suppresses MAPK and NF-κB signaling pathways, preventing cellular damage [17]. Caffeic acid phenethyl ester (CAPE), a naturally occurring derivative, inhibits histone acetyltransferases (HATs) and suppresses UVB-induced MMP-1 production in fibroblasts and keratinocytes [17]. Ferulic acid stabilizes Vitamin C and Vitamin E, enhancing their photoprotective efficacy and preventing UV-induced lipid peroxidation [19]. It protects human dermal fibroblasts against UVA irradiation by mitigating oxidative stress and inflammatory signaling, and prevents UVB-induced DNA damage. Ferulic acid also inhibits UVA and UVB-induced ROS production, reduces cytotoxicity, lipid peroxidation, and DNA alterations while protecting against degradation of heat-shock proteins [19,20]. Topical application reduces signs of photoaging, improving skin hydration, color, erythema, and topography. Additionally, it inhibits MMP-2 and MMP-9, preventing collagen degradation in the extracellular matrix [21,22]. Sinapic acid exhibits strong antioxidant and anti-inflammatory properties. In UVB-irradiated fibroblasts, it reduces ROS and MMP-1 production by inhibiting the MAPK/NF-κB pathway [17]. Canola meal extract containing sinapic acid decreases MMP-12 production and increases pro-collagen I alpha 1 content in neonatal fibroblasts exposed to UVB radiation [23].

#### 3.1.2. Curcumin

Curcumin, the active polyphenolic compound derived from the rhizome of *Curcuma longa* (turmeric), has garnered significant attention to combat photoaging. It has a variety of actions, such as antioxidant, anti-inflammatory, and anti-collagenase properties [24,25]. Hwang et al. examined the effects of curcumin on the expression of matrix metalloproteinases MMP-1 and MMP-3 in human dermal fibroblast cells. Their Western blot analysis showed that curcumin inhibited the UVB-induced expression of MMP-1 and MMP-3. Additionally, curcumin significantly reduced the production of reactive oxygen species (ROS) caused by UVB exposure in fibroblasts. The researchers found that curcumin also inhibited the UVB-induced activation of nuclear factor (NF)-κB and activator protein (AP)-1. Moreover, curcumin effectively suppressed the UVB-induced phosphorylation of p38 and c-Jun N-terminal kinase, and prevented UVB-induced MMP expression by blocking the mitogen-activated protein kinase/NF-κB pathway and AP-1 activation [25]. Zhan et al. developed a curcumin-loaded liposome formulation embedded in a sodium alginate gel to enhance curcumin’s bioavailability and investigate its protective role against photodamage. They assessed the antioxidant properties, drug diffusion efficiency, and therapeutic effects of the formulation. In mouse model studies, liposomal curcumin exhibited strong antioxidant activity in DPPH and hydrogen peroxide assays. Under UV exposure, it effectively scavenged free radicals produced by lipid peroxidation in the skin, serum, and liver, and boosted superoxide dismutase levels. Overall, the findings suggest that liposomal curcumin can help prevent UV-induced epidermal and cellular damage [24]. Chen et al. investigated how curcumin protects against skin damage caused by UVB radiation. Researchers used both in vivo (mouse) and in vitro (HaCaT keratinocyte) models to elucidate the underlying molecular mechanisms. Chen et al. discovered that curcumin treatment significantly reduced skin damage, normalized tissue structure, and lowered inflammatory marker levels. In vitro experiments showed that curcumin restored viability, decreased apoptosis, reduced ROS levels, and preserved mitochondria. Curcumin increased markers of mitochondrial autophagy (PINK1, Parkin, LC3-II) and reduced P62, indicating enhanced autophagic flux. Curcumin binds stably to YAP1, especially at Leu351, Asn354, Val357—enhancing mitochondrial protection [26,27,28,29,30].

### 3.2. Tannins

Tannins are often found in nuts, seeds, bark; they are divided into three groups: procyanidins, prodelphinidins, propelargonidins. The properties of proanthocyanidins vary. Proanthocyanidins from Pinus thunbergii, mainly catechin/epicatechin, show significant antioxidant properties. Proanthocyanidins from tea black currant, cranberry, grapes, bilberry, pine bark, peanut skin decrease ROS, induce lower iNOS and COX-2 expression, lower inflammation, have anticancer properties, and are effective against many diseases. Among procyanidins, anti-inflammatory and antioxidant properties have been proven to be presented by grape seed procyanidins, in addition to the antitumor properties of procyanidin B1 [31]. ANPs, areca nut procyanidins, upon being tested in CD-1 mice, proved to be effective against UVB-induced skin photoaging. UVB-induced skin of the mice, after oral intake of areca nut procyanidins, presented less significant macroscopic changes such as epidermal thickness and collagen disorientation than in non-treated mice, as well as inhibited expression of COX-2, MMP-2, MMP-9 and TIMP [32]. In an in vitro experiment, influence of tannic acid on UVB-irradiated L929 fibroblasts was researched. Tannic acid was proven to effectively scavenge radicals, as well as inhibit callagenase, elastase, MMP-1 expression alongside NADH oxidase activation, ROS production and lipid peroxidation [33]. Methanol extracts of *Dipterocarpus tuberculatus* Roxb containing high amounts of tannin were used to determine the antiphotoaging effect on UVB-induced normal human dermal fibroblasts and nude mice. It was proven to inhibit UVB-induced changes in NO concentration, SOD activity, Nrf2 expression, MMP-2, MMP-9, COX-2 in fibroblasts, and macroscopic changes in nude mice [34]. UVB-irradiated human epidermal keranocytes treated with gallotannin were proven to have improved collagen synthesis, reduced metalloproteinase-1 (MMP-1) expression in a dose-dependent manner, and downregulated MMP-1 levels through the ERK/JNK signaling pathway [35].

### 3.3. Flavonoids

Flavonoids are a diverse group of polyphenolic compounds known for their antioxidant, anti-inflammatory, and anti-photoaging effects [36]. They are further classified into several subgroups.

#### 3.3.1. Isoflavones

Isoflavones include genistein, which is found in soybeans (*Glycine max*) and inhibits kinase activation, prevents UV-induced inflammation, reduces MMP-1 expression, and enhances collagen synthesis, preserving skin elasticity [37]. Genistein exhibited protective effects in UVB-irradiated HDFs by inhibiting apoptosis, decreasing MDA levels, and reducing FKHRL1 and p66Shc activity while increasing SOD levels [38]. Soy-derived aglycones also exhibit strong antioxidant properties, enhancing cellular defense against oxidative stress [39]. Coumestrol, a metabolite of daidzein (an isoflavone from soybeans, *Glycine max*), demonstrated potent anti-photoaging effects by inhibiting UVB-induced MMP-1 expression. It exerted this effect through the suppression of FLT3 kinase activity in a three-dimensional human skin equivalent model [40].

#### 3.3.2. Flavan-3-ols

Flavan-3-ols are widely recognized for their antioxidant properties. Among flavan-3-ols the most representative compounds are catechins. Green tea (*Camellia sinensis*) is a rich source of catechins, comprising approximately 30% of the dry weight of tea leaves. The major compounds include catechin (C), epicatechin (EC), epigallocatechin (EGC), epicatechin-3-gallate (ECG), epigallocatechin-3-gallate (EGCG), and gallocatechin-3-gallate (GCG). Their antioxidant activity is attributed to the presence of dihydroxy and trihydroxy groups on the B-ring, with additional enhancement from the gallate moiety found in ECG, EGCG, and GCG [36].

Green tea catechins counteract UV-induced skin damage through multiple mechanisms. EGCG, the most abundant catechin, has been shown to inhibit tyrosinase activity, thereby reducing melanin production in UV-exposed melanocytes. EGCG-treated α-MSH-stimulated melanoma cells exhibited suppressed tyrosinase and MITF (microphthalmia-associated transcription factor) expression, which are key regulators of pigmentation. EGCG inhibits tyrosine kinase activity of the epidermal growth factor receptor (EGFR), preventing ROS formation and collagen degradation. It also scavenges ROS, and prevents elastase activity, maintaining skin elasticity and reducing wrinkle formation [41]. EGCG also exerts potent anti-wrinkle effects by binding to collagenase, preventing its interaction with free collagen chains. It suppresses UV-induced MMP production and collagenase activity, thereby preserving collagen integrity. In vivo studies on hairless mice confirmed EGCG’s ability to prevent UV-induced degradation of the extracellular matrix (ECM) and inhibit elastase activity [36]. The antioxidant effects of EGCG are also well-documented. It effectively scavenges ROS, enhances the activity of superoxide dismutase (SOD) and glutathione peroxidase (GSH-Px), and inhibits MAP kinase signaling pathways. Co-application of EGCG with vitamin C was shown to enhance photodamage repair [36].

In UVB-irradiated HaCaT cells, EGCG treatment (especially in nanoparticle form) led to reduced ROS levels, decreased MDA concentration, and inhibition of MMP-2 and MMP-9 expression [36,41]. In another study, EGCG reduced apoptosis and suppressed c-Fos, p21, and p53 mRNA expression, as well as TNF-α and IL-6 secretion. EGCG administration in UVB-irradiated HDFs further demonstrated its protective effects by inhibiting collagen degradation, reducing MMP-13, MMP-8, and MMP-1 production, and suppressing MAPK, ERK1/2, p38 MAPK, and JNK phosphorylation. Topical application of EGCG in rats exposed to UVA radiation resulted in macroscopically visible improvements, including a reduction in sunburn cells and dermo-epidermal activation [38].

Additionally, catechin application in UVA-irradiated rats led to increased CAT, GPx, and SOD levels while decreasing TBARS concentrations, indicating enhanced antioxidant defense [38].

#### 3.3.3. Flavonols

Flavonols include quercetin, a compound found in various fruits and vegetables (f.ex. red onion—*Allium cepa*, tomato—*Solanum lycopersicum*), and especially in *Aloe vera*; quercetin directly targets JAK2 and PKCδ, preventing UV-induced photoaging by inhibiting MMP-1 production and pro-inflammatory cytokines. Quercetin also enhances collagen synthesis and protects the extracellular matrix from UV degradation [17]. Myricetin, a flavonol, has been shown to exhibit protective effects against UV-induced skin damage. Pretreatment of UVB-irradiated primary human keratinocytes (PHKCs) with myricetin led to a decrease in malondialdehyde (MDA) and hydrogen peroxide (H_2_O_2_) levels, as well as the suppression of JNK activation. In vivo studies on UVB-irradiated hairless mice treated with myricetin-conjugated beads showed a reduction in epidermal thickening and inhibition of MMP-9 production and Raf enzyme activity, leading to improved skin structure and reduced inflammation [42]. Another flavonol, fisetin, exhibited anti-photoaging properties in UVB-irradiated human dermal fibroblasts (HDFs) by reducing collagen degradation, inhibiting MMP-9, MMP-3, MMP-1, COX-2, nitric oxide (NO), and PGE2 expression. Fisetin also suppressed ROS generation and inhibited the activation of JNK, ERK, and CREB pathways [43].

#### 3.3.4. Flavones

Baicalin, derived from the roots of *Scutellaria lateriflora Georgi*, has demonstrated significant photoprotective properties. In UVB-induced human dermal fibroblasts (HDFs), baicalin administration resulted in the suppression of MMP-1 expression. Additionally, another study reported a decrease in the expression of TGF-β1, GPx, SOD, and MDA levels, along with reduced expression of p16, p53, p66, tissue inhibitor of metalloproteinase (TIMP-1), and MMP-1 mRNA. Furthermore, levels of TNF-α and IL-6 were also reduced [44]. In vivo studies on UVB-irradiated mice showed that topical application of baicalin led to a significant reduction in skin thickening, hyperplasia, and edema. It also decreased the expression of MMP-1, MMP-3, and MMP-9, as well as COX-2, IL-1β, IL-10, and iNOS. Additionally, baicalin treatment promoted increased collagen I and III production, highlighting its potential in preventing and reversing photoaging [44].

#### 3.3.5. Flavanones

Among flavanones one of the attention-deserving examples is hesperitin, derived mainly from grapefruit (*Citrus* × *paradise*) and lemon (*Citrus* × *limon*). Hesperetin administration to HDFs before UVB irradiation, as well as to hairless mice before UVA exposure, resulted in reduced MMP-1 activity and collagen degradation. Additionally, it downregulated Nrf2 activity and the expression of NQO-1 and GST, which are crucial for antioxidant defense [44]. Hesperidin, another flavanone, exhibited significant antioxidant and anti-inflammatory effects. In UVA-irradiated HaCaT cells, hesperidin increased SOD activity while reducing IL-1β, IL-6, and TNF-α expression. Oral administration of hesperidin to hairless mice prior to UVB irradiation led to a decrease in wrinkle formation, collagen fiber loss, and transepidermal water loss (TEWL). Additionally, it suppressed MMP-9, IL-8, TNF-α, MEK, and ERK expression, further reinforcing its protective role against photoaging [45].

Naringenin, another potent flavanone, mainly present in and derived from grapefruit (*Citrus* × *paradise*), demonstrated anti-photoaging effects in vitro and in vivo. Pre-administration of naringenin to HaCaT cells before UVB irradiation resulted in the suppression of MMP-1, AP-1, FRA1, and ERK2 expression. In hairless mice, oral administration of naringenin reduced wrinkle formation, TEWL, and MMP-13 expression, while topical application inhibited the production of IL-1β, IL-10, IL-6, and TNF-α [46].

#### 3.3.6. *Aloe vera*

*Aloe vera* (AV) is often regarded as a remarkable natural remedy. The genus comprises around 550 species, and both the leaves and roots are rich in bioactive phytochemicals such as aloe-emodin, aloin, and quercetin. These compounds are commonly used in both food products and traditional herbal medicine worldwide. AV is well known for its therapeutic effects, particularly its immunomodulatory, anti-inflammatory, and antioxidant activities, making it especially valuable in treating skin conditions, supporting aesthetic dermatology, and aiding tissue repair [27,28]. Sun et al. investigated the protective effects of exosome-like nanoparticles derived from Aloe vera gel and rind—referred to as gADNPs and rADNPs—against ultraviolet (UV)-induced skin photoaging. In both in vivo and in vitro models, the two types of nanoparticles demonstrated excellent biocompatibility and the ability to delay skin photoaging. These effects were mediated through the activation of the Nrf2/ARE pathway to combat oxidative stress. The treatment significantly inhibited the overproduction of ROS, decreased pro-inflammatory cytokines (TNF-α and TGF-β), and improved cell migration and proliferation. In parallel, UVA-irradiated dermal fibroblasts (DFs) pretreated with these nanoparticles showed reduced mitochondrial ROS production, lower levels of apoptosis, and restoration of critical anti-aging markers such as collagen type I (COL1A1) and LaminB1. In vivo experiments on ICR mice exposed to combined UVA and UVB radiation showed that both gADNPs and rADNPs effectively alleviated signs of skin photoaging, such as erythema, dryness, and wrinkling. Histological staining revealed that epidermal thickening was significantly reduced, collagen and elastic fibers were restored, and inflammatory infiltration was suppressed [28]. Lee et al. demonstrated that lactic acid fermentation of Aloe vera leaf skin significantly enhances its anti-aging effects on UVB-damaged human skin fibroblasts, primarily by restoring mitochondrial function. The fermented extract, rich in quercetin and other bioactives from Aloe vera, showed superior efficacy compared to hot water extracts or quercetin alone. AV notably improved mitochondrial enzyme activity, reduced oxidative stress, and promoted collagen synthesis while inhibiting MMP-1 expression, which contributes to wrinkle formation. These findings suggest that antioxidant and mitochondrial-protective properties of the fermented extract make it a promising ingredient for cosmeceutical applications, especially using Aloe processing byproducts [29]. A study by Misawa et al. investigated the protective effects of Aloe vera-derived sterols against chronic UVB-induced skin photoaging in hairless mice. For seven weeks, mice were given diets with different amounts of Aloe vera gel extract (AVGE) and had their skin exposed to UVB radiation. The results demonstrated that oral AVGE treatment considerably decreased UVB-induced skin damage, including reduced skin dryness, wrinkles, and epidermal thickness. Aloe sterols decreased UV-induced apoptosis in skin cells and maintained dermal collagen fibers, according to histological research. Additionally, pro-inflammatory cytokines (IL-1β and TNF-α) and matrix metalloproteinases (MMP-2, MMP-9, MMP-12, and MMP-13), which are important mediators of collagen degradation and wrinkle formation, were inhibited by aloe sterol therapy. According to these results, aloe sterols help to preserve the structure and function of the skin under UV damage by regulating MMP and having anti-inflammatory properties [30].

### 3.4. Stilbenes

Stilbenes, a group of polyphenols based on 1,2-diphenylethylene, consist of resveratrol, picetannol and pterostilbene. As a group, they show anti-inflammatory, antiviral, anticancer and antioxidant activities.

#### 3.4.1. Resveratrol

Resveratrol (3,5,4’-trihydroxy-trans-stilbene) exists in two isomeric forms: cis-resveratrol, which is unstable, and trans-resveratrol, which, despite its stability, can convert into the cis-isomer under UV exposure or high pH. The main source of this compound consists of the skin of grapes (*Vitis*), blueberries (*Vaccinium caesariense*), raspberries (*Rubus idaeus*) and mulberries (*Morus nigra*). Resveratrol upregulates antioxidant enzyme activity and scavenges free radicals [47]. It is known for its potent antioxidant, anti-inflammatory, and anticancer properties. It scavenges ROS, inhibits MMPs, and enhances collagen synthesis, protecting against UV-induced skin aging and hyperpigmentation. Resveratrol also modulates the skin’s immune response by stimulating anti-inflammatory cytokines and inhibiting pro-inflammatory cytokines [48,49].

#### 3.4.2. Piceatannol

Piceatannol (3,4,3’,5’-tetrahydroxy-trans-stilbene), found in various fruits, such as grapes (*Vitis*), passion fruit (*Passiflora edulis*), as well as palm seeds *Aiphanes horrida*, and even wine, is a metabolite of resveratrol with superior anticancer properties. It inhibits protein-tyrosine kinase activity and exhibits low water solubility and bioavailability. Piceatannol has been shown to mitigate oxidative damage in lung tissues, promote collagen synthesis, suppress fatty acid-induced oxidative stress, and activate the Nrf2/NQO1 pathway [50].

#### 3.4.3. Pterostilbene

Pterostibene, a dimethyl ester derivative of resveratrol, extracted from blueberries (*Vaccinium caesariense*) and grapevines (*Vitis*), has demonstrated strong antioxidant and anti-inflammatory properties. It has been studied for its role in managing skin disorders, as well as its anticancer properties, which include inducing necrosis, apoptosis, and autophagy of cancerous cells [51].

### 3.5. Lignans

Lignans such as sesamin and silymarin possess antioxidant, anti-inflammatory, and antitumor properties. They protect against UV-induced oxidative stress and collagen degradation by inhibiting MMPs and enhancing collagen synthesis [52].

#### 3.5.1. Sesamin

Sesamin, a lignan derived from *Sesamum indicum* seeds, exhibits strong antioxidant and anti-inflammatory properties. It enhances collagen type I expression and increases alkaline phosphatase activity [53].

#### 3.5.2. Silymarin

Silymarin a complex of lignans derived from *Silybum marianum*, consists of silybin, isosilybin, silydianin, silychristin, and 2,3-dehydrosilybin (DHSB). These compounds absorb UV light (peaking at ~325 nm), providing an additional layer of UV protection. Silybin effectively inhibits elastase, collagenase, and hyaluronidase activity, with the strongest effects observed for DHSB. In vitro studies demonstrated that silybin reduces UVA-induced caspase-3 activity, as well as MMP-1, HO-1, and HSP70 production [54,55].

#### 3.5.3. Coumarins

Coumarin is a lactone (benzopyrone) known for its sweet, vanilla-like odor and bitter taste. It has been reported to exhibit anti-inflammatory, antioxidant, and anticancer properties. Naturally occurring in cinnamon, vanilla grass, sweet woodruff, sweet clover, and tonka bean, coumarins represent an important group of bioactive compounds with diverse therapeutic potential [56].

#### 3.5.4. Urolithin A

Urolithin A is a benzo-coumarin derived from gut microbiota (rather than plants); it has demonstrated significant anti-UVA-induced photoaging activity in fibroblasts. It inhibits extracellular matrix degradation, suppresses senescence-associated enzyme activity, and reduces reactive oxygen species (ROS) accumulation. This reduction in ROS leads to increased NRF2 translocation and subsequent activation of antioxidative enzymes, enhancing cellular defense mechanisms against oxidative stress [57].

#### 3.5.5. Decursin

Decursin, a coumarin derivative extracted from the roots of *Angelica gigas*, is widely known for its anticancer properties. In research focused on photoaging, decursin was found to inhibit UVB-induced MMP-1 and MMP-3 expression while also activating nuclear factor-κB (NF-κB), a key regulator of inflammatory responses [58]. Additionally, recent studies have demonstrated that decursin inhibits melanogenesis in UVB-irradiated melanocytes by downregulating tyrosinase and tyrosinase-related protein activity [59].

#### 3.5.6. Umbelliferone

Umbelliferone (7-hydroxycoumarin, 7-OHC), an isomer of caffeic acid derived from carrots (*Daucus carota*), mouse-ear hawkweed (*Hieracium pilosella*) and bigleaf hydrangea (*Hydrangea macrophylla*), has demonstrated antioxidant, anti-inflammatory, and free radical scavenging properties. It has been reported to downregulate TGF-beta1 in kidney tissue, activate Nrf2-mediated HO-1 expression in hepatotoxicity models, and increase intracellular ROS levels in cancer cells (human oral carcinoma KB) [60]. 7-OHC has demonstrated antioxidant effects in UVB-irradiated fibroblasts by inhibiting NF-κB, COX-2, MMP-1, and MMP-9 expression while increasing the activity of antioxidant enzymes [61]. This suggests its potential role in photoprotection and anti-aging skincare formulations.

#### 3.5.7. Hydrangenol

Hydrangenol (dihydroisocoumarin), obtained from *Hydrangeaceae* leaves, exhibits antifungal, anti-inflammatory, antiallergic, antidiabetic, and anti-angiogenic activities. In vitro experiments on UVB-irradiated fibroblasts revealed that hydrangenol suppresses the expression of MMP-1, MMP-3, hyaluronidase (HYAL-1 and HYAL-2), COX-2, IL-6, IL-8, and IL-1β, while also reducing ROS production [62]. Additionally, in vivo studies on UVB-irradiated hairless mice showed that oral administration of hydrangenol reduced wrinkle formation, epidermal thickness, and transepidermal water loss (TEWL) in a dose-dependent manner. At the molecular level, hydrangenol upregulated the expression of involucrin, filaggrin, and aquaporin-3 (AQP3), which were inhibited by UVB exposure. It also increased hyaluronic acid (HA) and collagen type I production while downregulating UV-induced COX-2, IL-6, MMP-1, and MMP-3 expression. Further investigation revealed that hydrangenol inhibits UVB-induced activation of the STAT1 and MAPK signaling pathways by reducing the phosphorylation of AP-1, p38, and ERK while promoting Nrf2/ARE pathway activation. This process enhances the expression of antioxidative stress proteins such as HO-1, NQO-1, GCLM, and GCLC [62,63]. These findings support hydrangenol’s potential as an anti-photoaging agent.

#### 3.5.8. Esculetin

Esculetin (6,7-dihydroxycoumarin), derived from Chinese ash (*Fraxinus chinensis*), has been shown to reduce acute and chronic topical skin inflammation [64,65]. It has been found to significantly reduce MMP-1 production in UVB-irradiated fibroblasts and exhibit similar activity in keratinocytes [61,65].

#### 3.5.9. Scopoletin

Scopoletin (6-methoxy-7-hydroxycoumarin), a compound commonly used in traditional Chinese medicine (*Artemisia capillaris* crude extract), has been noted for its antioxidant, anti-inflammatory, and immunomodulatory properties. In a study where fibroblasts were exposed to conditioned medium from UVB-irradiated keratinocytes, treatment with scopoletin led to decreased MMP-1, IL-1α, and TNF-α mRNA production. It also inhibited MAPK and nuclear factor-κB (NF-κB) pathways, reducing cytotoxicity and signs of senescence in fibroblasts [66,67].

### 3.6. Cannabinoids

Cannabinoids are naturally occurring compounds found in *Cannabis sativa*, known for their biological activities relevant to skin health and condition. They interact with the endocannabinoid system (ECS), which includes cannabinoid receptors (CB1 and CB2), endocannabinoids (2-AG and AEA), and enzymes involved in their synthesis and degradation. The ECS plays a crucial role in maintaining skin homeostasis, regulating immune responses, and protecting against oxidative stress. Cannabinoids have gained significant interest for their potential in treating photoaging and other skin conditions due to their ability to modulate inflammation, oxidative stress, and collagen degradation [68,69].

#### 3.6.1. Cannabidiol (CBD)

Cannabidiol (CBD) is a non-psychoactive cannabinoid known for its potent anti-inflammatory and antioxidant properties. It interacts with CB1 and CB2 receptors, as well as other targets such as transient receptor potential (TRP) channels and peroxisome proliferator-activated receptors (PPARs), influencing inflammation, cell proliferation, and oxidative stress regulation [69,70]. CBD inhibits NF-κB activation and TNF-α production, reducing oxidative stress and preventing apoptosis. It also activates the Nrf2/ARE pathway, enhancing the production of antioxidant enzymes such as heme oxygenase-1 (HO-1), superoxide dismutase (SOD), and catalase (CAT). By modulating these pathways, CBD effectively reduces ROS levels, protects against lipid peroxidation, and preserves the integrity of cellular membranes [70]. In a study on UV-irradiated keratinocytes, CBD was shown to reduce ROS production, inhibit COX-2 expression, and suppress the production of pro-inflammatory cytokines such as IL-6 and TNF-α. Additionally, CBD enhanced the production of the anti-inflammatory lipid mediator lipoxin A4 (LXA4), reducing inflammation and promoting skin barrier repair [70]. Łuczaj et al. demonstrated that topical application of CBD reversed UV-induced disruptions in cellular membrane composition by decreasing lysophospholipids and increasing sphingomyelin production. This preserved membrane integrity and prevented oxidative damage in both keratinocytes and fibroblasts, highlighting CBD’s protective role against UV-induced photoaging [71]. In another study, CBD prevented the UV-induced degradation of collagen by stimulating enzymes (hospholipase A2) involved in collagen cross-linking and maintaining the extracellular matrix’s structural integrity. This suggests that CBD not only protects against oxidative stress and inflammation but also preserves collagen and elastin, preventing wrinkle formation and loss of elasticity [71,72].

#### 3.6.2. Cannabigerol (CBG)

Cannabigerol (CBG) is another non-psychoactive cannabinoid known for its antioxidant and anti-inflammatory properties. CBG interacts with CB2 receptors and modulates TRP channels, influencing inflammatory responses and melanin synthesis. It also activates PPAR receptors, contributing to its anti-proliferative and anti-inflammatory effects [73]. CBG has been shown to reduce ROS production and inhibit MMP-1 expression, protecting collagen from UV-induced degradation. In keratinocytes, CBG prevented oxidative damage by preserving antioxidant enzyme activity and maintaining mitochondrial function. Additionally, CBG inhibited the activation of NF-κB and MAPK signaling pathways, reducing the production of pro-inflammatory cytokines and preventing chronic inflammation [73]. In a study on UVB-irradiated skin cells, CBG was found to protect against oxidative DNA damage, reduce lipid peroxidation, and prevent apoptosis. It also enhanced cellular viability and reduced UV-induced erythema, demonstrating its photoprotective potential against photoaging. CBG’s antioxidant and anti-inflammatory effects were attributed to its ability to modulate TRPV1 channels and PPAR-γ activity, reducing oxidative stress and inflammation at the molecular level [73]. Both CBD and CBG exhibit strong anti-inflammatory and antioxidant properties, making them promising candidates for preventing and treating photoaging [71]. They protect against UV-induced oxidative stress, reduce inflammation, and preserve collagen and elastin, preventing wrinkle formation and loss of skin elasticity. Their ability to interact with the ECS and modulate multiple signaling pathways provides a unique advantage in maintaining skin homeostasis and preventing chronic inflammation. The use of CBD and CBG in skincare products is gaining popularity due to their safety profile and efficacy in preventing photoaging. Topical formulations containing CBD and CBG offer targeted photoprotection by neutralizing ROS, reducing pro-inflammatory cytokines, and preserving the extracellular matrix. Additionally, advanced delivery systems, such as nanoparticles and liposomes, enhance their bioavailability and penetration into the deeper layers of the skin, increasing their efficacy [71,72,74]. However, further clinical studies are needed to fully understand the long-term effects and optimal dosages of CBD and CBG in photoaging prevention. Research on their synergistic effects with other antioxidants and anti-inflammatory agents could lead to more effective formulations for photoprotection and skin rejuvenation.

### 3.7. Vitamins

Vitamins are essential micronutrients known for their potent antioxidant, anti-inflammatory, and collagen-synthesizing properties, making them effective agents in preventing and treating photoaging. They protect against UV-induced oxidative stress, enhance skin barrier function, and promote collagen synthesis, preserving skin elasticity and preventing wrinkle formation. This section focuses on vitamin C, vitamin A, and vitamin E and their role in photoaging prevention and skin rejuvenation [75,76].

#### 3.7.1. Vitamin C (L-Ascorbic Acid)

Vitamin C, also known as L-ascorbic acid (L-AA), is a potent water-soluble antioxidant essential for maintaining skin health and preventing photoaging. Main sources of this compound include blackcurrant (*Ribes nigrum*) brussel sprouts (*Brassica oleracea*), *Malpighia emarginata*, kakadu plum (*Terminalia ferdinandiana*) and many more. It acts as a reductant by donating electrons, neutralizing free radicals, and preventing oxidative damage to cellular components such as proteins, lipids, and DNA. During this process, Vitamin C is oxidized into ascorbyl radical, which is further converted into dehydroascorbic acid and eventually reduced back to active ascorbate—the biologically active form responsible for its antioxidant effects [76]. Vitamin C plays a critical role in collagen synthesis, acting as a cofactor for ferrous and 2-oxoglutarate-dependent dioxygenases, which are essential for proline and lysine hydroxylation, stabilizing the collagen triple helix. It stimulates the transcription of procollagen type I and III genes, enhancing collagen production and maintaining the extracellular matrix’s structural integrity. Vitamin C also inhibits activator protein-1 (AP-1), which is activated by UV-induced ROS, thereby preventing MMP production and collagen degradation [75]. Topical application of Vitamin C has shown significant improvements in skin condition, including a reduction in deep wrinkles, enhanced elasticity, and improved hyperpigmentation. In clinical trials, vitamin C inhibits tyrosinase activity, thereby reducing melanin synthesis. This effect was particularly effective in reducing UV-induced hyperpigmentation, leading to a more even skin tone [77,78]. Vitamin C also enhances the skin’s photoprotective properties by stabilizing Vitamin E, regenerating the alpha-tocopherol radical back to its active form, and preventing lipid peroxidation. This synergistic effect between Vitamin C and Vitamin E provides enhanced antioxidant protection against UV-induced oxidative stress and photoaging [78]. Despite its potent antioxidant properties, Vitamin C is highly unstable and prone to oxidation when exposed to light, heat, and air. This instability poses challenges in formulating effective topical applications. Additionally, its hydrophilic nature limits its ability to penetrate the lipid barrier of the stratum corneum, reducing its bioavailability and efficacy in the deeper layers of the skin [75]. To overcome these challenges, several advanced delivery systems have been developed to enhance Vitamin C’s stability, penetration, and sustained release. One approach involves the use of hydrophobic Vitamin C derivatives, such as magnesium ascorbyl phosphate (MAP) and ascorbyl-6-palmitate, which can diffuse through the lipid barrier and be enzymatically converted into active Vitamin C within cells [75]. Another strategy is the use of nanoparticles, including liposomes and solid lipid nanoparticles, which encapsulate Vitamin C, protecting it from oxidation and enhancing its penetration into the deeper layers of the skin. These nanoparticles also provide controlled release, ensuring a steady supply of Vitamin C for prolonged antioxidant protection and collagen synthesis [77]. Additionally, reverse micelles in oil/water/oil emulsions have been developed to entrap Vitamin C and improve its stability and delivery. These formulations create a protective environment for Vitamin C, reducing its degradation and enhancing its transdermal absorption [75]. Topical application of Vitamin C has been shown to improve skin hydration, elasticity, and firmness while reducing wrinkles, fine lines, and hyperpigmentation. Clinical studies have demonstrated its efficacy in reducing UV-induced erythema, preventing oxidative DNA damage, and enhancing the skin’s natural barrier function [75]. Combining Vitamin C with other antioxidants, such as Vitamin E and ferulic acid, further enhances its photoprotective and anti-photoaging effects. This synergistic combination stabilizes Vitamin C, enhances its penetration, and provides broad-spectrum antioxidant protection against UVA and UVB radiation [79].

#### 3.7.2. Retinoids

Retinoids are a class of chemical compounds derived from Vitamin A (retinol) known for their potent anti-aging, anti-inflammatory, and collagen-synthesizing properties. They regulate cell proliferation, differentiation, and apoptosis, influencing skin remodeling, pigmentation, and immune responses. Retinoids are widely used in dermatology and cosmetology for treating photoaging, acne, hyperpigmentation, and other skin conditions. They are classified into natural retinoids (retinol, retinaldehyde, and retinoic acid) and synthetic retinoids (adapalene, tazarotene, and alitretinoin). Retinoids are found in many plants, such as sweet potato (*Ipomoea batatas*) or carrots (*Daucus carota).* This section focuses on the mechanisms of action, therapeutic potential, and clinical applications of all-trans-retinoic acid (ATRA) and bakuchiol, a natural retinoid analogue [80,81,82].

#### 3.7.3. All-Trans-Retinoic Acid (ATRA)

All-trans-retinoic acid (ATRA) is considering the most active form of retinoid, known for its potent anti-aging and anti-photoaging properties. It is produced from retinol through oxidation to retinaldehyde and further conversion to ATRA by retinaldehyde dehydrogenase. ATRA is involved in cell proliferation, differentiation, apoptosis, and collagen synthesis. ATRA helps skin cells called keratinocytes grow faster, which speeds up skin renewal. Furthermore, it encourages fibroblasts to make more collagen types I and III. This helps rebuild the skin’s structure and keeps it elastic. Moreover, ATRA blocks enzymes called MMPs that break down collagen. It does this by lowering the activity of a protein called AP-1. This helps prevent wrinkles and collagen loss. Additionally, ATRA boosts the levels of a protein called TGF-β and activates a pathway called TGF-β/Smad. This increases collagen production and helps repair the skin. It also raises the amount of fibronectin and hyaluronic acid in the skin. These substances improve skin hydration, firmness, and flexibility. Together, these effects reduce fine lines, wrinkles, and sagging skin, making ATRA a powerful anti-aging treatment. ATRA also fights inflammation. It lowers the levels of inflammatory signals like IL-6 and TNF-α. It controls how immune cells like dendritic cells, T cells, and macrophages behave. This strengthens the immune system and helps prevent long-term inflammation linked to skin aging. ATRA is widely used in topical formulations for treating photoaging, acne, hyperpigmentation, and other skin conditions. It effectively reduces fine lines, wrinkles, and pigmentation by promoting collagen synthesis, epidermal renewal, and melanin dispersion. Clinical studies have demonstrated its efficacy in improving skin texture, elasticity, and brightness, reducing visible signs of aging. However, ATRA is associated with side effects such as irritation, dryness, erythema, and peeling, particularly during the initial phase of treatment. These adverse effects are attributed to its strong exfoliating and cell turnover properties. To minimize irritation, ATRA is often formulated in low concentrations or combined with moisturizers and anti-inflammatory agents. Gradual titration and nighttime application are recommended to enhance tolerance and efficacy [83].

#### 3.7.4. Bakuchiol

Bakuchiol is a natural meroterpene found in *Psoralea corylifolia seeds*, known for its retinol-like properties and potent antioxidant, anti-inflammatory, and anti-tumor effects. It is considered a plant-derived retinoid analogue due to its ability to modulate gene expression through retinoic acid receptors (RARs) and retinoid X receptors (RXRs), thereby promoting collagen synthesis, epidermal renewal, and pigmentation regulation. Bakuchiol is a promising alternative to retinol, offering similar anti-photoaging benefits without the irritation or photosensitivity associated with synthetic retinoids [84,85]. Bakuchiol enhances collagen synthesis by stimulating the production of types I, III, and IV collagen, promoting ECM remodeling and increasing skin elasticity. It also inhibits MMPs, preventing collagen degradation and preserving the structural integrity of the dermal matrix. In addition, bakuchiol increases the expression of elastin and fibronectin, improving skin firmness and resilience [84]. It enhances epidermal turnover, promoting the shedding of dead skin cells and stimulating the regeneration of new, healthy keratinocytes. This process leads to a brighter complexion, improved skin texture, and reduced hyperpigmentation [84,86]. Bakuchiol exhibits potent antioxidant properties by scavenging ROS and activating the Nrf2/HO-1 pathway, enhancing the cellular antioxidant defense system. It also reduces oxidative stress by inhibiting NADPH oxidase and preventing lipid peroxidation, protecting cellular components from UV-induced damage [84]. Bakuchiol’s anti-inflammatory effects are attributed to its ability to inhibit NF-κB activation and downregulate pro-inflammatory cytokines such as IL-1β, IL-6, and TNF-α. It also suppresses COX-2 and iNOS expression, reducing inflammation and preventing chronic inflammatory responses associated with photoaging [85]. Bakuchiol is well-tolerated and does not cause irritation or photosensitivity, making it suitable for sensitive skin types. Clinical studies have shown that bakuchiol effectively reduces fine lines, wrinkles, and pigmentation, improves skin elasticity and hydration, and enhances overall skin radiance. In a double-blind, randomized controlled trial, bakuchiol demonstrated comparable anti-aging effects to retinol without causing irritation, erythema, or peeling [85]. Bakuchiol is commonly used in topical formulations such as serums, creams, and lotions for anti-photoaging, anti-pigmentation, and overall skin rejuvenation. Its antioxidant and anti-inflammatory properties enhance its photoprotective effects, making it an effective agent for preventing and treating photoaging [86].

#### 3.7.5. Vitamin E

Vitamin E is a lipid-soluble antioxidant. It plays a pivotal role in combating photoaging. Vitamin E can be found in avocado (*Persea americana*), almond (*Prunus amygdalus*) and in many natural oils such as sunflower oil or hazelnut oil. Recent research highlights Vitamin E’s multifaceted protection mechanisms, including antioxidant action, anti-inflammatory properties, and anti-glycation effects. Markiewicz et al. [87] demonstrated that Vitamin E significantly inhibits reactive oxygen species (ROS) production and glycation-related damage in skin fibroblasts exposed to methylglyoxal. While it showed moderate collagen fiber preservation, its real value lies in reducing ROS accumulation and limiting crosslinking of collagen via glycation pathways—two key mechanisms present in developing photoaged skin. Fu et al. [88] investigated an innovative approach that combined Vitamin E with exosomes derived from human adipose stem cells (hADSC-Exos). This combination significantly reduced UVB-induced oxidative damage and extracellular matrix (ECM) breakdown in experimental skin models. Vitamin E notably contributed to greater collagen production and helped prevent thinning of the epidermis, indicating strong potential for use in regenerative anti-aging skin treatments [88]. Al-Rawi et al. conducted a comprehensive literature review and determined that Vitamin E acts as both a protective and reparative agent against UV-related skin damage. It helps maintain cellular membrane integrity, lowers lipid peroxidation, and suppresses UV-induced inflammation. Notably, applying 5% tocopherol topically before UV exposure significantly reduced wrinkle development and tumor incidence by up to 75% in animal models, although its effectiveness against UVA radiation was found to be less reliable. The authors also highlighted that advanced formulations—such as tocopherol sorbate and vitamin E phosphate—enhanced photoprotective outcomes, and that nanoparticle-based Vitamin E creams improved skin absorption and helped prevent radiation-induced dermatitis [89].

### 3.8. Carotenoids

Carotenoids are a group of lipophilic pigments found in fruits and vegetables such as sweet potato (*Ipomoea batatas*), carrots (*Daucus carota*) or spinach (*Spinacia oleracea*), known for their potent antioxidant, anti-inflammatory, and photoprotective properties. They are divided into two main categories: carotenes (non-polar hydrocarbons) and xanthophylls (polar and oxygenated derivatives). Carotenoids accumulate in the skin, particularly in the stratum corneum, where they protect against UV-induced oxidative stress, prevent collagen degradation, and maintain skin elasticity [90]. This section focuses on three key carotenoids: β-carotene, lycopene, and astaxanthin.

#### 3.8.1. β-Carotene

β-Carotene, found in carrots (*Daucus carota*) or spinach (*Spinacia oleracea*), is a precursor of Vitamin A and is known for its potent antioxidant and anti-inflammatory properties. It scavenges ROS, prevents lipid peroxidation, and protects cellular components from oxidative damage. β-Carotene also inhibits the expression of Intercellular Adhesion Molecule 1 (ICAM-1) and vascular cell adhesion molecule 1 (VCAM-1), reducing NF-κB activation and peroxynitrite levels, thereby preventing inflammation and oxidative stress. In a study on UV-irradiated skin cells, β-carotene was found to inhibit MMP-1, MMP-3, and MMP-10 production, preventing collagen degradation and maintaining the extracellular matrix’s structural integrity. Additionally, β-carotene reduced UVA-induced ROS production and protected against DNA damage by scavenging free radicals [91]. Clinical studies have shown that oral supplementation of β-carotene improves skin elasticity and hydration while reducing transepidermal water loss and wrinkle formation. However, high doses of β-carotene can lead to skin discoloration (carotenodermia), emphasizing the need for controlled dosage in photoaging prevention [92].

#### 3.8.2. Lycopene

Lycopene is a potent antioxidant carotenoid found in tomatoes (*Solanum lycopersicum*) and other red fruits such as grapefruits (*Citrus* × *paradisi*) or strawberry guava (*Psidium cattleyanum*). It is considered one of the most effective ROS scavengers among carotenoids due to its unique molecular structure with an extended conjugated double-bond system. Lycopene neutralizes singlet oxygen and peroxyl radicals, thereby protecting against UV-induced oxidative stress, lipid peroxidation, and DNA damage. [93]. Oral supplementation of lycopene has been shown to significantly reduce acute photodamage, potentially preventing long-term photoaging effects. A study demonstrated that lycopene supplementation reduced UV-induced erythema and sunburn cells, protecting the skin from DNA damage and apoptosis [94]. High concentrations of lycopene in the skin correlate with reduced wrinkle depth and smoother skin surface. Clinical studies have shown that lycopene improves skin texture, elasticity, and hydration, reducing visible signs of aging. In a randomized controlled trial, participants who consumed lycopene-rich tomato paste showed a significant reduction in UV-induced erythema and oxidative stress markers compared to the control group. Additionally, lycopene inhibits MMP-1 and MMP-9 expression, preventing collagen degradation and preserving the extracellular matrix’s structural integrity. It also downregulates pro-inflammatory cytokines such as IL-6 and TNF-α, reducing chronic inflammation and preventing photoaging [91,94].

#### 3.8.3. Astaxanthin

Astaxanthin is a xanthophyll carotenoid known for its potent antioxidant, anti-inflammatory, and collagen-preserving properties. It is produced by various microalgae, particularly *Haematococcus pluvialis*, and accumulates in the skin’s lipid bilayers, preserving membrane integrity and preventing oxidative damage. Astaxanthin’s unique molecular structure with a conjugated double-bond system and polar end groups enables it to span the cell membrane, effectively neutralizing ROS and protecting cellular components from oxidative stress [95]. Astaxanthin is considered the most potent antioxidant among carotenoids, exhibiting significantly higher ROS-scavenging activity than β-carotene and lycopene. It directly scavenges ROS and activates the Nrf2/HO-1 pathway, upregulating antioxidant enzymes such as superoxide dismutase (SOD), catalase (CAT), and glutathione peroxidase (GPX). This dual mechanism enhances the cellular antioxidant defense system, protecting proteins, lipids, and DNA from oxidative damage [74]. Astaxanthin inhibits UV-induced MMP-1, MMP-3, and elastase expression, preventing collagen and elastin degradation and maintaining skin elasticity. It also stimulates collagen synthesis by upregulating TGF-β expression, promoting ECM remodeling and reducing wrinkle formation [95,96]. In addition to its antioxidant and collagen-preserving effects, astaxanthin exhibits strong anti-inflammatory properties. It reduces UV-induced inflammation by inhibiting Inducible Nitric Oxide Synthase (iNOS), cyclooxygenase-2 (COX-2), and prostaglandin E2 expression in keratinocytes. Astaxanthin also downregulates pro-inflammatory cytokines such as interleukin-1β (IL-1β), interleukin-6 (IL-6), and tumor necrotizing factor-α (TNF-α), preventing chronic inflammation and protecting against photoaging [97,98]. Astaxanthin’s antitumor activity further enhances its photoprotective properties. It enhances the cytotoxic activity of natural killer (NK) cells and cytotoxic T lymphocytes, promoting an anti-tumor immune response. In murine models, astaxanthin was shown to stimulate antibody production and improve humoral immunity, reducing the risk of UV-induced skin cancer [96]. Clinical trials evaluating oral and topical supplementation of astaxanthin have shown significant improvements in skin condition, including reduced wrinkles, enhanced elasticity, and increased hydration [99]. Astaxanthin also reduces hyperpigmentation and age spots, improving skin tone and brightness. Singh et al. reported that astaxanthin supplementation decreased MMP-1 and MMP-12 mRNA levels while increasing protocollagen type I mRNA, highlighting its role in preventing collagen degradation and promoting collagen synthesis [98]. Astaxanthin is unique among carotenoids in that it does not convert into Vitamin A, making it safe for long-term use without the risk of hypervitaminosis A. Its high stability and broad-spectrum antioxidant activity make it an ideal candidate for preventing photoaging and maintaining skin health [95]. 

## 4. Discussion

The growing incidence of skin cancer and the rising global awareness of skin health have led to an intensified scientific focus on developing effective strategies for preventing skin aging, particularly photoaging. With the booming skincare and aesthetic medicine industries—estimated to be worth billions of dollars worldwide—the need for safe, effective, and scientifically validated anti-photoaging solutions has never been greater.

**Figure 2:**
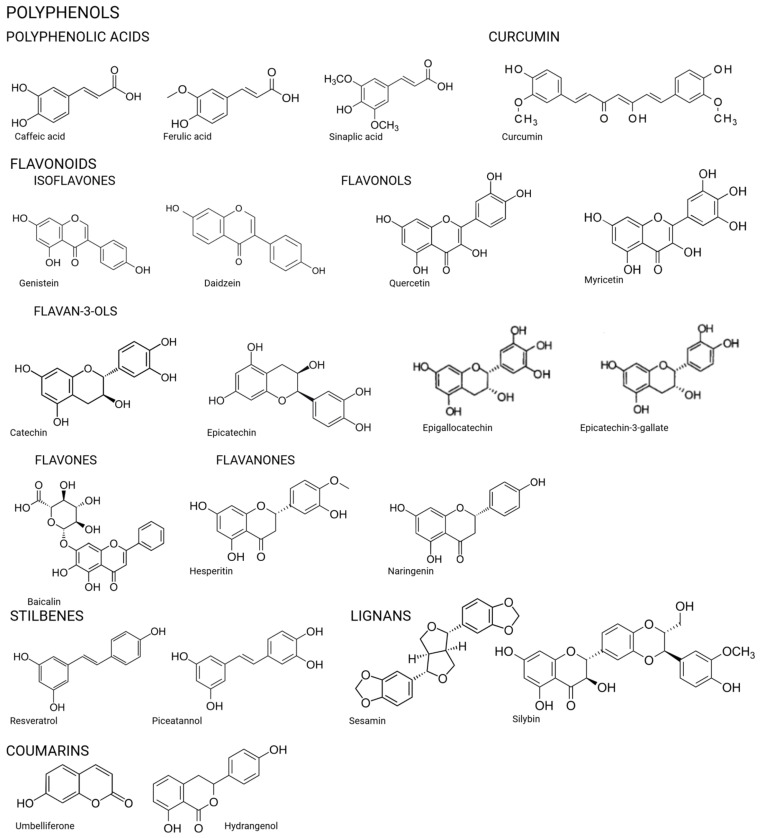

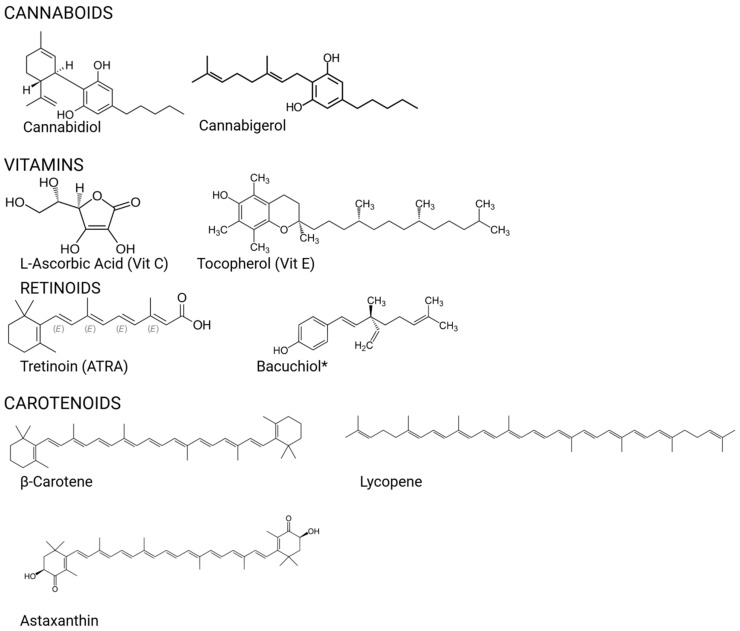
Chemical structures of the compounds.

Skin aging, especially photoaging, is a multifaceted process driven by prolonged UV exposure, oxidative stress, and inflammation, leading to structural and functional changes in the skin. Natural compounds (Figure 2) have emerged as effective alternatives to synthetic agents for combating these effects, offering diverse mechanisms of action. Among the most promising are marine-derived bioactive peptides, which have shown significant potential in mitigating photoaging by reducing oxidative stress, inflammation, MMP expression, and excessive melanin synthesis. These properties position them as promising candidates for addressing the physiological alterations associated with photoaging [10,11,100]. Similarly, plant-derived extracts such as polyphenols (e.g., curcumin and aloe vera) and flavonoids with many in vivo and clinical trials have demonstrated potent antioxidant and anti-inflammatory effects, protecting against UVB-induced oxidative stress and DNA damage [13,15]. Another effective natural compound is coffee extract, rich in polyphenols, flavonoids, and caffeine, which has been shown to reduce oxidative stress, improve skin elasticity, and combat inflammation [93,94]. Among these compounds, astaxanthin stands out as one of the most potent and versatile agents for combating photoaging. Unlike other compounds, astaxanthin has been shown to exert its effects across multiple pathways, combining antioxidant, anti-inflammatory, and collagen-preserving activities all at once. Clinical studies have demonstrated that oral supplementation with astaxanthin significantly improves skin elasticity, reduces transepidermal water loss, and downregulates MMP expression, thereby preventing collagen degradation and wrinkle formation [95,96,97]. In topical formulations, astaxanthin has proven highly effective in reducing UVB-induced oxidative stress, inflammation, and structural skin damage, while preserving skin hydration and barrier integrity. Additionally, advanced delivery systems such as nanoparticle encapsulation have enhanced its cellular uptake and antioxidant activity, further increasing its efficacy. Animal studies have also shown that astaxanthin cream decreases MMP-1 expression and increases type I procollagen levels, underscoring its ability to protect skin integrity at the molecular level [95,98].

Carotenoids, including β-carotene, lycopene, and astaxanthin, have been widely studied for their ability to preserve collagen, reduce inflammation, and neutralize reactive oxygen species (ROS), further contributing to photoprotection [90,91].

To conclude, extensive experimental and clinical evidence from many diverse studies supports the efficacy of natural products’ success in protection against skin aging (Table 1). All the studies discussed in this review reveal the benefits of the used substances without side effects.

## 5. Conclusions

By summarizing the most recent and relevant in vitro and in vivo findings, this review aims to support the development of innovative, safe, and effective strategies for skin photoprotection and anti-aging therapy. The article highlights the latest research into plant-derived substances that offer protection against UV-induced skin damage.

In conclusion, the field of photoaging prevention is rapidly evolving, supported by both the public’s demand for aesthetic solutions and the medical need for effective skin cancer prevention. Continued research, especially well-designed clinical trials, is essential to confirm the safety, efficacy, and therapeutic relevance of these substances and their derivatives. The agents reviewed in this paper represent a hopeful future for integrative, evidence-based skincare and photoaging prevention strategies.

## Figures and Tables

**Table 1:** Bioactive compounds with anti-photoaging properties.

Compound	Source	Mechanism	Mode of Action	Effects	Concentration	References
Polyphenols						
Caffeic Acid	Coffee, fruits	Prooxidant/antioxidant depending on context	Induces ROS, inhibits MMP-1, MAPK & NF-κB pathways	Anticancer, anti-photoaging, UV protection	35, 87 µM [16] 2.5, 5 µM [17] 100, 200 µM [18]	[16,17,18]
Ferulic Acid	Grains, fruits	Antioxidant, anti-inflammatory	Scavenges ROS, inhibits NF-κB, stabilizes vit. C/E	Reduces wrinkles, pigmentation	40 μg/mL [20] 14% [21,22]	[20,22]
Curcumin	Curcuma longa	Antioxidant, anti-inflammatory	Inhibits NF-κB, AP-1, MAPK; restores mitochondrial autophagy	Reduces ROS, inhibits MMP-1/3, UVB protection	4 mg/mL [24] 10, 30 µM [25] 5, 10 µM [26]	[24,25,26]
Tannins	Nuts, seeds	Antioxidant, anti-inflammatory	Inhibits MMPs, COX-2	Reduces UV damage, improves structure	10 µg/mL [50]	[50]
Genistein (Isoflavone)	Soy	Antioxidant, kinase inhibitor	Inhibits MMP-1, enhances collagen synthesis	Maintains elasticity, prevents inflammation	10 μmol/L [33]	[32,33]
EGCG (Flavan-3-ol)	Green tea	Potent antioxidant	Inhibits tyrosinase, MMPs, EGFR	Anti-wrinkle, photoprotection, elasticity	7.0–13.0 mg/g [31] 5,10,20 µM [35]	[31,35,36]
Quercetin (Flavonol)	Fruits & vegetables	JAK2/PKCδ inhibitor	Inhibits MMP-1, enhances collagen synthesis	Prevents ECM degradation	2.5 μM and 5 μM [17]	[17,36]
Myricetin	Berries	Antioxidant	Inhibits MMPs, MAPKs	Prevents collagen degradation	1, 5 nmol [37]	[37]
Baicalin (Flavone)	Scutellaria lateriflora	Antioxidant, MMP inhibitor	Inhibits MMPs, ILs, COX-2, promotes collagen	UV protection, anti-inflammatory	25 μg/mL [39]	[39]
Hesperidin (Flavanone)	Citrus fruits	Antioxidant	Inhibits MMP-9, MAPK, ILs, TNF-α	Skin hydration, reduces wrinkles	100 mg/kg [40]	[40]
Aloe Vera	Aloe	Antioxidant, immunomodulator	Activates Nrf2/ARE, reduces ROS, inflammation	Anti-wrinkle, collagen restoration, UV protection	50, 500 mg/kg/day [27] 1.103 μg/μL [28] 0.1–0.5% [29] 20 μg/mg [30]	[27,28,29,30]
**Stilbenes**						
Resveratrol (Stilbene)	Grapes, berries	Antioxidant, anti-inflammatory	Scavenges ROS, enhances collagen	Anti-photoaging, anti-hyperpigmentation	4.24 μM [42] 1.46 μg/cm^2^ [43] 0.5 mg/kg; 0.5 mL/100 g [44]	[42,43,44]
Piceatannol (Stilbene)	Grapes, passion fruit	Antioxidant, kinase inhibitor	Activates Nrf2/NQO1	Anti-photoaging, collagen synthesis	10–25 μM [45]	[45]
**Coumarins**						
Hydrangenol	Hydrangea	Antioxidant, anti-inflammatory	Inhibits MMPs, COX-2, increases HA	Reduces wrinkles, improves moisture	5, 10, 20, 40 mg/kg [56]	[56]
Vitamin C	Fruits, vegetables	Antioxidant	Scavenges ROS, regenerates vit. E, inhibits AP-1	Stimulates collagen, reduces pigmentation	10% [73]	[70,73]
Carotenoids	Fruits, vegetables	Antioxidant, UV absorber	Scavenges ROS, inhibits MMPs	Improves elasticity, reduces TEWL	10.6 mg per day [85] 6,12 µmol/L [86] 24 mg/day [89]	[85,86,89]
Astaxanthin	Algae	Antioxidant, anti-inflammatory	Activates Nrf2/HO-1, inhibits MMPs, COX-2	Reduces wrinkles, improves moisture	10, 20 μg/cm^2^ [91] 4, 8 μM [92] 0.055–1.3 mg/L [93]	[90,91,92,93]
Bakuchiol	Psoralea corylifolia	Retinol-like	Stimulates collagen, antioxidant	Improves elasticity, reduces wrinkles	5 µg/mL [81]	[80,81]

## Data Availability

All data are available in the manuscript.
